# Supplementary material for: Relationship between social cognition and emotional markers and acoustic-verbal hallucination in youth with post-traumatic stress disorder: Protocol for a prospective, 2-year, longitudinal case-control study
Source: PLoS One. 2024 Jul 2;19(7):e0306338. doi: 10.1371/journal.pone.0306338 (PMC11218996; doi:10.1371/journal.pone.0306338)
Supplement: S1 File — (PDF) [file pone.0306338.s002.pdf]

Child psychiatry and multidisciplinary research (public health, psychodynamics, neuroscience and human and social sciences) dedicated to children exposed to the Nice attack on July 14, 2016

Program 14-7

MSA n°4 - Amendment n°4 to protocol V0.0

of 07/01/2020

TABLE OF MODIFICATIONS/JUSTIFICATIONS

Developer

Children's hospitals CHU-LENVAL

57, Avenue de la Californie - 06200 Nice

Tel : 04 92 03 03 92 ; Fax : 04 92 03 03 44

fondation@lenval.com

And by delegation of promotion management:

Nice University Hospital

Department of Clinical Research and Innovation Hôpital de

Cimiez

4, av Reine Victoria

BP1179 06003 Nice cedex 01

Tel: 04 92 03 40 11 - Fax: 04 92 03 40 75

drc@chu-nice.fr

COORDINATING INVESTIGATOR

Prof. Florence Askenazy

University Department of Child and Adolescent Psychiatry

Hôpitaux Pédiatriques de Nice CHU-Lenval

57 av de la Californie

06200 Nice

Tel : 04.92.03.04.39 - *Fax :* 04.92.03.04.43

e-mail: askenazy.f@pediatrie-chulenval-nice.fr

Old version of the protocol: V3.0 of 26/07/2019 New version of the protocol: V4.0 of 07/01/2020 Amendment n°4 final version: 0.0 of 07/01/2020

MODIFIED SECTIONS OF THE PROTOCOL AND RATIONALE, INFORMATION LEAFLET AND CONSENT

Previous version	Modified version	Justification
**Protocol cover page:**<br>Version N°3.0 du 26/07/2019	Version N°4.0 of 07/01/2020	Version and date change following amendment
**Page 38 of the protocol: Study rationale - Scientific background**<br>F/ The "physalis child" study	The "14-7 Program" is an opportunity to study, in a pediatric population, the impact of a very specific type I trauma of a mass attack. It also seems essential to extend our study to all types of trauma in children and adolescents with non-psychotic AVH, and not just the July 14, 2016 attack. Indeed, the clinical semiology of non-psychotic hallucinations describes a symptomatology that is transient and disappears spontaneously in 95% of cases (Garralda 1984a; Escher et al. 2004; Rubio et al. 2012), or an evolution towards a psychotic pathology in the event of persistent AVH (McGee, Williams, and Poulton 2000; Poulton R et al. 2000; Dhossche et al. 2002), both of which prove to be non-inclusion criteria for the current study. Furthermore, the literature describes a semiological difference in PTSD between mass and individual trauma types that would also be interesting to highlight in our study with regard to non-psychotic HAV (Djelantik et al. 2017; Malarbi et al. 2017; Gamache Martin, Van Ryzin, and Dishion 2016). The comparison of a group from the "14-7 Program" (Group 2) with a group presenting with PTSD following individual trauma (Group 5) would reinforce the work of clinical and psychopathological observation of PTSD in children and adolescents. This analysis, which would complement the results of our first study, would provide a better understanding of the psychopathology of the disorder, and optimize the treatment of children and adolescents with PTSD.<br>how to deal with this population.	**Clinical interest in** extending the origin of PTSD to any trauma in order to make a semiological comparison between mass trauma and individual trauma.<br><br>**Methodological interest in the feasibility of** patient inclusion. Non-psychotic AVH are mostly transient and benign. If they persist, they can lead to a diagnosis of psychosis (DSM 5). 3 years after the attack on 14.7, the presence of AVH in exposed patients certainly no longer fits the description of "non-psychotic AVH".

**Protocol page 47: Assumptions and objectives**<br><br>**B/ Objectives**<br>Primary and secondary objectives for the "Physalis child" study	The primary objective is to identify social and emotional cognition factors related to the presence of non-psychotic AVH in the cohort of children exposed to the mass trauma of July 14, 2016 in Nice, but also to any type of individual trauma, compared to a cohort of children with PTSD without non-psychotic AVH. Secondary objectives are:<br>- reassess the evolution of non-psychotic HAV at 6 months, 1 year and 2 years,<br>- identify social and emotional cognition factors related to the persistence of non-psychotic AVH in the cohort of children exposed to the mass trauma of July 14, 2016 in Nice, but also to any type of individual trauma, compared to a cohort of children with PTSD without non-psychotic AVH<br>- reassess the evolution of the psychiatric diagnosis at 6 months and 2 years<br>- to enable a possible correlation between the persistence of hallucinations and the new psychiatric diagnosis.<br><br>A first analysis of the data will allow a comparison between all children exposed to trauma (mass and individual) with non-psychotic HAV and children with PTSD without non-psychotic HAV. A second analysis will compare the results between the group of children exposed to mass trauma with HAV (group 2) and the group of children exposed to individual trauma with non-psychotic HAV (group 3).<br>HAV (group 5).	Extension of the inclusion criterion from mass traumatic event (14.7) to individual traumatic event.<br><br>Explanation of the data analysis due to the presence of this new group of included patients with PTSD resulting from individual trauma.
**Protocol page 50: Assessment criteria A/**<br><br>**Primary endpoint**	**group 5**: children not exposed to the attack on July 14, 2016 with one or more traumatic antecedents included in the "L'enfant physalis" study in Nice.	Description of the new patient group to be included. Group different from group 4 coach included in Nice

**Protocol page 59: Evaluation criteria B/**<br><br>**Secondary evaluation criteria**<br><br>B8/ Evaluation criteria for the "Physalis child" study<br>○ Screening for hallucinations<br><br>The screening questionnaire (Appendix 1) used in our original study comprises seven items. The questions are short and explicit, designed to be easily understood by children and adolescents.<br><br>It is based on the five items in the schizophrenia section of the Diagnostic Interview Schedule for Children-Child version (DISC-C) (Costello A, Edelbrock C, and Kalas R 1982).<br><br>Two items assess the existence of acoustic-verbal hallucinations. Acoustic-verbal hallucinations are defined by a positive response to the questions: "Have you ever heard a voice calling you by your first name or talking to you when no one else can hear it?" and "Have you ever heard your dolls or toys talking to you, answering you when you play with them?". Only patients<br>(item C) are included.	The question to screen for the presence of non-psychotic hallucination is taken from the Adolescent Dissociative Experiences Scale II (A-DES) (Armstrong et al. 1997) "I hear voices in my head that aren't mine" and the K-SADS-PL (Kaufman et al. 2016)<br>"Do you hear voices that no one else can hear?<br><br>The screening question posed to the non-psychotic child or adolescent for inclusion in the study is: *"Do you hear a voice that is different from yours and that no one else can hear?"*<br><br>The question posed in this way makes it possible to better discriminate auditory perceptual disorders without eliminating the question of auditory reliving specific to PTSD, which is also the focus of the study.	Simplification of the question asked, enabling the screening of auditory perceptual disorders and thus making it possible to include HAV and auditory reliving linked to PTSD.
**Page 60 of the protocol: Evaluation criteria**<br><br>**B/ Secondary evaluation criteria**<br><br>B8/ Evaluation criteria for the "Physalis child" study<br>○ Standardized categorical clinical assessment	The MINI-Kid 1.1 (Sheehan et al. 2010) explores, in a standardized way, the main psychiatric disorders of the DSM-5 axis in children and adolescents aged 8 to 16. The interview is divided into 20 modules, each corresponding to a diagnostic category. The diagnosis of post-traumatic stress disorder is listed according to the DSM-5 and the	A clarification is made, as the document is not the same depending on whether the subject included is a child or an adolescent.

The MINI-Kid 1.1 (Sheehan et al. 2010) explores, in a standardized way, the main psychiatric disorders of the DSM-5 axis in children and adolescents aged 8 to 16. The interview is divided into 20 modules, each corresponding to a diagnostic category. The diagnosis of post-traumatic stress disorder is listed according to DSM-5 and ICD-10. There is a "parent" and a "child" version of the MINI-.<br>Kid 1.1.	ICD-10. There is a "parent" version and a "child" version.<br>"MINI-Kid 1.1.	
**Protocol page 85 :**<br><br>**10-The "Physalis child" study A/**<br><br>**Selection of subjects**<br><br>• Study population<br><br>The study population corresponded to patients included in the "Programme 14-7" within the Service Universitaire de Psychiatrie de l'Enfant et de l'Adolescent of the Hôpitaux Pédiatriques de Nice CHU-Lenval, (Pr Askenazy).<br><br>In this cohort of patients with PTSD, subjects with non-psychotic hallucinations and control subjects with no hallucinations were screened, matched for sex and age.<br><br>Two groups were thus formed: a case group, called "Acoustic-Verbal Hallucination" (AVH) +, and a control group, called "AVH -". The performance of a	The study population corresponded to child and adolescent patients, aged 8 to 16, without a diagnosis of psychosis (DSM 5) and presenting with a diagnosis of PTSD related to mass trauma (patients included in the "14-7 Program") (group 2) or individually (group 5).<br><br>In this cohort of PTSD patients, we screened subjects with non-psychotic hallucinations and control subjects without hallucinations, matched for sex and age (+/- 6 months).<br><br>Two groups are thus formed: a group of cases, called the<br>"Acoustic-Verbal Hallucination (AVH) +" group, and a control group, called the<br>"HAV -". A case-control study was carried out to understand why, in patients with the same clinical diagnosis of	Redefinition of the target population extended to all types of trauma.<br><br>Matching clarification<br><br>Clarification given concerning the management of data used to differentiate the population exposed to the

case-control study attempted to understand why, among patients presenting the same clinical picture of PTSD, some reported the presence of non-psychotic hallucinations and others not.	PTSD, some reported the presence of non-psychotic hallucinations, while others did not. Within each of the two groups, a differentiation will also be made by the type of trauma (individual or mass) at the time of the event. through a binary categorical variable.	mass trauma or individual trauma.
**Page 85 of the protocol :**<br><br>**10-The "Physalis child" study A/**<br><br>**Selection of subjects**<br><br>• Inclusion criteria<br><br>HAV + group<br><br>- Age greater than or equal to 8 and strictly less than 16 at inclusion<br><br>- With HAV (self-administered screening questionnaire)<br><br>- With a diagnosis of PTSD (MINI-kid 1.1)<br><br>- Affiliated with a social security scheme ;<br><br>- Good command of the French language (francophone)<br><br>Children whose parents have agreed to participate in the study (informed consent)<br><br>Groupe HAV -<br><br>- Age greater than or equal to 8 years and strictly	HAV + Group<br><br>- Age greater than or equal to 8 and strictly less than 16 at inclusion<br><br>- With HAV (screening questionnaire)<br><br>- With a diagnosis of PTSD (K-SADS-PL "TPST" section)<br><br>- Affiliated with a social security scheme ;<br><br>- Good command of the French language (francophone)<br><br>- Children whose parents have agreed to participate in the study (informed consent)<br><br>Groupe HAV -<br><br>- Age greater than or equal to 8 and strictly less than 16 at inclusion<br><br>- No HAV (screening questionnaire)<br><br>- With a diagnosis of PTSD (K-SADS-PL "TPST" section)<br><br>- Affiliated with a social security scheme ;	Adaptation of the text to reflect changes in the tools used to screen for AVH. The diagnosis of PTSD is made using the K-SADS-PL and the MINI-kid 1.1 for better integration of the study.<br>The "physalis child" in phase II of the "14.7" protocol for groups 2, 4 and 5.

under 16 years of age at inclusion<br><br>- Without HAV (screening self-questionnaire)<br><br>- With a diagnosis of PTSD (MINI-kid 1.1)<br><br>- Affiliated with a social security scheme ;<br><br>- Good command of the French language (francophone)<br><br>Children whose parents have agreed to participate in the study (informed consent)	- Good command of the French language (francophone)<br><br>- Children whose parents have agreed to participate in the study (informed consent)	
**Page 86 of the protocol :**<br><br>**10-The "Physalis Child" study A/**<br><br>**Selection of subjects**<br><br>• Non-inclusion criteria<br><br>- Intellectual disability (IQ below 70)<br><br>- Genetic, neurological or neurosensory pathologies<br><br>- Child or adolescent with a psychotic or autistic disorder (MINI-kid 1.1 and "psychosis" section of the K-SADS-PL)	- Intellectual disability (IQ below 70)<br>- Genetic, neurological or neurosensory pathologies<br>- Child or adolescent with a psychotic disorder (section "psychosis" section of K-SADS-PL) or autism (autism spectrum disorder section of MINI-Kid 1.1).	Adaptation of the text in connection with the modification of the tools used to diagnose psychotic disorder using the K-SADS-PL and autistic disorder using the MINI-kid 1.1 for better integration of the "L'enfant physalis" study in phase II of the "14.7" protocol for groups 2, 4 and 5.

**Page 87 of the protocol**<br><br>**10-The "Physalis child" study C/**<br><br>**Conduct of the study**<br><br>• Intervention<br><br>All patients with PTSD included were from the "Program 14-7". The HAV screening self-questionnaire will be offered to subjects included in group 2, aged 8 to 16, in addition to the questionnaires carried out as part of the "Programme 14-7".<br><br>Subjects with non-psychotic AVH identified by the self-questionnaire make up the<br>The "HAV +" (case) group and the "HAV -" (control) group comprise non-psychotic HAV-free subjects matched to the "HAV +" group according to sex and age.<br><br>Case and control subjects are recruited after the child or adolescent and his or her family have been informed, and the patient and parents (or representative of parental authority) have signed consent forms.<br><br>The inclusion visit (T0) is the first stage in the assessment process, during which the inclusion criteria are verified by means of scales administered as part of the<br>"Program 14-7:<br><br>- Results of the self-questionnaire for acoustic-verbal hallucinations	The study will be offered to any patient with PTSD. A simple HAV screening question will be asked of the subjects included, aged between 8 and 16.<br><br>Subjects whose non-psychotic HAVs have been screened make up the "HAV +" (case) group, while the "HAV -" (control) group comprises non-psychotic HAV-free subjects matched to the "HAV +" group according to sex and age.<br><br>Case and control subjects are recruited after the child or adolescent and his or her family have been informed, and the patient and parents (or representative of parental authority) have signed consent forms.<br><br>The inclusion visit (T0) is the first stage in the evaluation process, during which the inclusion criteria are verified by means of scales administered as part of the "14-7 Program":<br><br>- Results of the acoustic-verbal hallucination screening questionnaire<br>- Diagnosis of PTSD by completing the "PTSD" section of the K-SADS-PL,<br><br>- Elimination of psychotic and autistic disorders through K-SADS-PL ("psychosis" section) and MINI-Kid tests 1.1 (autism spectrum disorder section)<br><br>- Absence of mental retardation assessed using the Wechsler Intelligence Scale for Children (WISC-V).<br><br>At the end of this assessment, for patients who meet the inclusion criteria and have given their agreement to take part in the study, a second assessment phase will enable the other tasks to be completed.	Adaptation of the text in line with the extension of the subjects included for all trauma (mass and individual) as well as the modification of the tools used for a better integration of the "Physalis child" study taking place in phase II of the protocol.<br>"14.7" for groups 2, 4 and 5.

- Diagnosis of TPST by MINI- Kid 1.1,<br>- Elimination of the diagnosis of psychotic and autistic disorders through MINI-kid 1.1 and K-SADS-PL ("psychosis" section),<br>- Absence of mental retardation assessed using the Wechsler Intelligence Scale for Children (WISC-V).<br><br>At the end of this assessment, for patients corresponding to the inclusion criteria and having given their agreement to take part in the study, a second assessment phase will enable the other specific study tasks to be completed. This assessment can take place during the same interview, following verification of the inclusion criteria, or during a second interview, at the subject's discretion.<br><br>This second assessment phase includes :<br><br>- Socio-demographic and clinical data collection<br>- Passage of scales allowing :<br>o Assessment of social cognition (NEPSY II)<br>o Determining the emotional profile (EED IV)<br>o The study of the emotional experience of acoustico-verbal hallucinations (BAVQ- R), only for patients with acoustico-verbal hallucinations, was carried out.	specific to the study. This evaluation can take place during the same interview, following verification of the inclusion criteria, or during a second interview, at the subject's discretion.<br><br>This second assessment phase includes :<br><br>- Socio-demographic and clinical data collection<br>- MINI-Kid 1.1 to assess comorbidities associated with PTSD: thymitic disorders, anxiety disorders, behavioral disorders, addiction.<br>- Passage of scales allowing :<br>o Assessment of social cognition (NEPSY II)<br>o Determining the emotional profile (EED IV)<br>o Study of the emotional experience of acoustic-verbal hallucination (BAVQ-R), only for patients with AVH)<br>-<br><br>At six months (T1), "HAV +" and "HAV -" patients were reviewed during a scheduled interview. These were:<br><br>- Evaluate the disappearance or persistence of auditory-verbal hallucinations by repeating the screening self-questionnaire.<br>- Repeat the MINI-Kid 1.1 or K-SADS-PL ("PTSD" and "psychosis" sections) to identify any change in the psychiatric diagnosis initially selected.<br><br>At one year (T2), the "14-7 Program" provides for a telephone recall of all subjects included in the study. The self-screening questionnaire for HAV can then be carried out over the telephone to assess the	

verbal)<br><br>At six months (T1), "HAV +" and "HAV -" patients were reviewed during a scheduled interview. These were:<br><br>- Evaluate the disappearance or persistence of the acoustic-verbal hallucinations by repeating the screening self-questionnaire.<br>- Repeat the MINI-Kid 1.1 or K-SADS-PL ("psychosis" section) to identify changes in the initial psychiatric diagnosis.<br><br>At one year (T2), the "14-7 Program" will call back all subjects included in the study. At this point, the AVH self-screening questionnaire can be administered by telephone, in order to assess the persistence of non-psychotic AVH.<br><br>Two years (+/- 6 months) after inclusion (T3), the "Program 14-7" provides for a new screening of the population where it will once again be possible :<br>- Evaluate the disappearance or persistence of the acoustic-verbal hallucinations by repeating the screening self-questionnaire.<br>- Repeat the MINI-Kid 1.1 or K-SADS-PL ("psychosis" section) to identify changes in the initial psychiatric diagnosis.<br>- Re-take the NEPSY II, from	persistence of non-psychotic HAV.<br><br>Two years (+/- 6 months) after inclusion (T3), the "14-7 Program" provides for a new screening of the population where it will again be possible :<br>- Evaluate the disappearance or persistence of auditory-verbal hallucinations by repeating the screening self-questionnaire.<br>- Repeat the MINI-Kid 1.1 or K-SADS-PL ("PTSD" and "psychosis" sections) to identify any change in the psychiatric diagnosis initially selected.<br>- Re-take the NEPSY II, EED IV and BAVQ-R (in subjects with persistent AVH), to assess changes in social and emotional cognition after PTSD.<br><br>The study ends for a patient when he or she has completed the various tasks. At the end of the study, patients continue their usual child psychiatric follow-up.<br><br>The study is taking place over a 3-year period, with an 18-month inclusion period and re-evaluation, for each patient included, at 6 months, 1 year and 2 years.<br><br>Modification of table 1 and figure 1	

EED IV and BAVQ-R (in subjects with persistent AVH), to assess changes in social and emotional cognition after PTSD.<br><br>The study ends for a patient when he or she has completed the various tasks. At the end of the study, patients continue their usual child psychiatric follow-up.<br><br>The study is taking place over a 3-year period, with an 18-month inclusion period and re-evaluation, for each patient included, at 6 months, 1 year and 2 years.		
**Information and consent notice L'enfant Physalis V3.0 of 26/07/2019**<br>**Example NI intended for the holder of parental authority**<br>**P1 :**<br>Your child was involved in the events that took place on July 14, 2016, in Nice, either directly (presence on the promenade, for example) or indirectly (presence of a family member, for example).<br><br>**Aim of the study: The** aim of this study was to detect the presence or absence of hallucinatory symptoms in a child who had been involved in the Nice attacks.	**<u>Version 4.0 of 07/01/2020</u>**<br><br>Addition of the notion of individual trauma other than the attack on July 14, 2016. :<br><br>Your child was involved in the events of July 14, 2016, in Nice, either directly (presence on the promenade, for example) or indirectly (presence of a family member, for example), or was involved in other traumatic events.<br><br>**Aim of the study: The** aim of this study was to detect the presence or absence of hallucinatory symptoms in children who had been involved in the Nice attacks or in any type of individual trauma.	Version and date change following amendment<br><br>Add characteristics of patients involved in an individual traumatic event<br><br>The same types of changes have been made to the notices patient information
