# Supplementary material for: Relationship between social cognition and emotional markers and acoustic-verbal hallucination in youth with post-traumatic stress disorder: Protocol for a prospective, 2-year, longitudinal case-control study
Source: PLoS One. 2024 Jul 2;19(7):e0306338. doi: 10.1371/journal.pone.0306338 (PMC11218996; doi:10.1371/journal.pone.0306338)
Supplement: S2 File — (PDF) [file pone.0306338.s003.pdf]

Recherche pédopsychiatrique et pluridisciplinaire (santé publique, psychodynamique, neurosciences et sciences humaines et sociales) consacrée aux enfants exposés à l'attentat de Nice le 14 juillet 2016

Programme 14-7

MSA n°4 - Amendement n°4 au protocole

V0.0 du 07/01/2020

TABLEAU DES MODIFICATIONS/JUSTIFICATIONS

Promoteur

Hôpitaux pédiatriques CHU-LENVAL

57, Avenue de la Californie – 06200 Nice

Tel : 04 92 03 03 92 ; Fax : 04 92 03 03 44

fondation@lenval.com

Et Par délégation de la gestion de la promotion:

CHU de Nice

Département de la Recherche Clinique et de l'Innovation

Hôpital de Cimiez

4, av Reine Victoria

BP1179 06003 Nice cedex 01

Tel: 04 92 03 40 11 – Fax: 04 92 03 40 75

drc@chu-nice.fr

INVESTIGATEUR COORDONNATEUR

Pr Florence Askenazy

Service Universitaire de Psychiatrie de l'Enfant et de l'Adolescent

Hôpitaux Pédiatriques de Nice CHU-Lenval

57 av de la Californie

06200 Nice

Tel : 04.92.03.04.39 - *Fax :* 04.92.03.04.43

e-mail: askenazy.f@pediatrie-chulenval-nice.fr

Ancienne version du protocole : V3.0 du 26/07/2019
Nouvelle Version du protocole : V4.0 du 07/01/2020
Amendement n°4 version finale : 0.0 du 07/01/2020

SECTIONS MODIFIEES DU PROTOCOLE ET JUSTIFICATION, NOTICE D'INFORMATION ET CONSENTEMENT

Version précédente	Version modifiée	Justification
**Page couverture du protocole:**<br>Version N°3.0 du 26/07/2019	Version N°4.0 du 07/01/2020	Changement de version et date suite à l'amendement
**Page 38 du protocole: Justificatif de l'étude – Contexte scientifique**<br>F/ L'étude « L'enfant physalis »	Le « Programme 14-7 » est l'occasion d'étudier, dans une population pédiatrique, l'impact d'un traumatisme de type I très particulier d'un attentat de masse. Il apparait également indispensable d'étendre notre étude à tous les type de trauma de l'enfant et l'adolescent présentant des HAV non psychotiques et non pas seulement celui de l'attentat du 14 juillet 2016. En effet, la sémiologique clinique des hallucinations non psychotiques décrit une symptomatologie transitoire et disparaissant spontanément dans 95% des cas (Garralda 1984a; Escher et al. 2004; Rubio et al. 2012) ou une évolution vers une pathologie psychotique en cas de persistance des HAV (McGee, Williams, and Poulton 2000; Poulton R et al. 2000; Dhossche et al. 2002), ce qui s'avère, dans les deux cas, être des critères de non inclusion pour l'étude actuelle. De plus, la littérature décrit une différence sémiologique du TSPT entre les type de traumatisme de masse et individuel qu'il serait également intéressant de mettre en évidence dans notre étude au sujet des HAV non psychotiques (Djelantik et al. 2017; Malarbi et al. 2017; Gamache Martin, Van Ryzin, and Dishion 2016). La comparaison d'un groupe issu du « Programme 14-7 » (groupe 2) avec un groupe présentant un TSPT à la suite d'un traumatisme individuel (groupe 5) renforcerait le travail d'observation clinique et psychopathologique du TSPT chez l'enfant et l'adolescent. Cette analyse, complémentaire aux résultats de notre première étude, permettrait ainsi d'accéder à une meilleure compréhension de la psychopathologie du trouble et d'optimiser la conduite à tenir auprès de cette population.	**Intérêt clinique** d'étendre l'origine du TSPT à tout trauma afin de réaliser un comparatif sémiologique entre le trauma de masse et le trauma individuel<br><br>**Intérêt méthodologique de faisabilité** dans l'inclusion des patients. Les HAV non psychotiques sont majoritairement transitoires et bénignes. En cas de persistances elles favorisent l'évolution vers un diagnostic de psychose (DSM 5). A 3 ans de l'attentat du 14.7, la présence d'HAV chez les patients exposés ne répondent certainement plus à la description des « HAV non psychotiques »

**Page 47 du protocole: Hypothèses et objectifs**<br><br>**B/ Les objectifs**<br>Objectifs principal et secondaires pour l'étude « L'enfant physalis »	L'objectif principal est d'identifier des facteurs de cognition sociale et émotionnelle liés à la présence d'HAV non psychotique au sein de la cohorte des enfants exposés au traumatisme de masse du 14 juillet 2016 à Nice, mais également à tout type de traumatisme individuel, comparé à une cohorte d'enfant présentant un TSPT sans HAV non psychotique. Les objectifs secondaires sont :<br>- de réévaluer l'évolution des HAV non psychotiques à 6 mois, 1 an et 2 ans,<br>- d'identifier des facteurs de cognition sociale et émotionnelle liés à la persistance d'HAV non psychotique au sein de la cohorte des enfants exposés au traumatisme de masse du 14 juillet 2016 à Nice, mais également à tout type de traumatisme individuel, comparé à une cohorte d'enfant présentant un TSPT sans HAV non psychotique<br>- de réévaluer l'évolution du diagnostic psychiatrique à 6 mois et 2 ans<br>- de permettre une éventuelle corrélation entre la persistance des hallucinations et le nouveau diagnostic psychiatrique.<br><br>Une première analyse des données permettra une comparaison entre l'ensemble des enfants exposés à un traumatisme (de masse et individuel) avec HAV non psychotique et des enfants avec TSPT sans HAV non psychotique. Une deuxième analyse permettra de comparer les résultats entre le groupe d'enfants exposés au traumatisme de masse avec HAV (groupe 2) et le groupe d'enfant exposés au traumatisme individuel avec HAV (groupe 5).	Extension du critère d'inclusion de l'événement traumatique non plus uniquement de masse (14.7) mais également individuel.<br><br>Explication de l'analyse des données du fait de la présence de ce nouveau groupe de patient inclus présentant un TSPT issu d'un trauma individuel.
**Page 50 du protocole: Critères d'évaluation**<br><br>**A/ Critère d'évaluation principal**	**groupe 5** : enfants non exposés à l'attentat du 14 juillet 2016 avec un ou plusieurs antécédent(s) traumatique(s) inclus à Nice dans le cadre de l'étude « L'enfant physalis »	Description du nouveau groupe de patient à inclure. Groupe différent du groupe 4 car inclus à Nice

**Page 59 du protocole: Critères d'évaluation**<br><br>**B/ Critères d'évaluation secondaires**<br><br>B8/ Critères d'évaluation de l'étude « L'enfant physalis »<br>○ Dépistage des hallucinations<br><br>Le questionnaire de dépistage (Annexe 1), utilisé dans notre étude princeps, comporte sept items. Les questions sont courtes et explicites, élaborées pour être facilement compréhensibles pour des enfants et adolescents.<br><br>Il est repris les cinq items figurant dans la section schizophrénie du Diagnostic Interview Schedule for Children-Child version (DISC-C) (Costello A, Edelbrock C, and Kalas R 1982).<br><br>Deux items évaluent l'existence des hallucinations acoustico-verbales. Les hallucinations acoustico-verbales sont définies par une réponse positive à la question : « As-tu déjà entendu une voix t'appeler par ton prénom ou te parler alors que personne d'autre ne l'entend ? » et « As-tu déjà entendu tes poupées ou tes jouets te parler, te répondre quand tu joues avec eux ? ». Seuls les patients cotant positivement (item C) sont inclus.	La question permettant de dépister la présence d'hallucination non psychotique est issue des échelles Adolescent Dissociative Expériences Scale II (A-DES) (Armstrong et al. 1997) « j'entends des voix dans ma tête qui ne sont pas les miennes » et de la K-SADS-PL (Kaufman et al. 2016) « entends-tu des voix que personne d'autre ne peut entendre ? ».<br><br>La question de dépistage posée à l'enfant à l'enfant ou l'adolescent non psychotique permettant de l'inclure dans l'étude est : *« Entends-tu une voix différente de la tienne et que personne d'autre ne peut entendre ? »*<br><br>La question posée ainsi permet de mieux discriminer les troubles perceptifs auditifs sans éliminer la question des reviviscences auditive propres au TSPT faisant également l'objet de l'intérêt de l'étude.	Simplification de la question posée permettant le dépistage des troubles perceptifs auditifs et permettant ainsi d'inclure les HAV et les reviviscences auditives liées au TSPT.
**Page 60 du protocole: Critères d'évaluation**<br><br>**B/ Critères d'évaluation secondaires**<br><br>B8/ Critères d'évaluation de l'étude « L'enfant physalis »<br>○ Evaluation clinique standardisée catégorielle	La MINI-Kid 1.1 (Sheehan et al. 2010) explore de façon standardisée les principaux troubles psychiatriques de l'axe du DSM-5 chez les enfants et adolescents de 8 à 16 ans. Il s'agit d'un entretien divisé en 20 modules, chacun correspondant à une catégorie diagnostique. Le diagnostic de trouble du stress post traumatique y est répertorié selon le DSM-5 et la	Une précision est apportée car il ne s'agit pas du même document selon si le sujet inclus est un enfant ou un adolescent.

La MINI-Kid 1.1 (Sheehan et al. 2010) explore de façon standardisée les principaux troubles psychiatriques de l'axe du DSM-5 chez les enfants et adolescents de 8 à 16 ans. Il s'agit d'un entretien divisé en 20 modules, chacun correspondant à une catégorie diagnostique. Le diagnostic de trouble du stress post traumatique y est répertorié selon le DSM-5 et la CIM-10. Il existe une version « parents » et une version « enfant » de la MINI-Kid 1.1.	CIM-10. Il existe une version « parents » et une version « enfant/adolescent » de la MINI-Kid 1.1.	
**Page 85 du protocole :**<br><br>**10-L'étude « L'enfant Physalis »**<br><br>**A/ Sélection des sujets**<br><br>• Population de l'étude<br><br>La population de l'étude correspond à des patients inclus dans le « Programme 14-7 » au sein du Service Universitaire de Psychiatrie de l'Enfant et de l'Adolescent des Hôpitaux Pédiatriques de Nice CHU-Lenval, (Pr Askenazy).<br><br>Il s'agit de dépister, au sein de cette cohorte de patient avec un TSPT, des sujets présentant des hallucinations non psychotiques et les sujets témoins ne présentant pas d'hallucinations, appariés selon le sexe et l'âge.<br><br>Deux groupes sont ainsi constitués : un groupe de cas, appelé « Hallucination Acoustico-Verbales » (HAV) + », et un groupe témoin, appelé « HAV - ». La réalisation d'une	La population de l'étude correspond à des patients enfants et adolescents, âgés de 8 à 16 ans, sans diagnostic de psychose (DSM 5) et présentant un diagnostic de TSPT en lien avec un traumatisme de masse (patients inclus dans le « Programme 14-7 ») (groupe 2) ou individuel (groupe 5).<br><br>Il s'agit de dépister, au sein de cette cohorte de patient avec un TSPT, des sujets présentant des hallucinations non psychotiques et les sujets témoins ne présentant pas d'hallucinations, appariés selon le sexe et l'âge (+/- 6mois).<br><br>Deux groupes sont ainsi constitués : un groupe de cas, appelé « Hallucination Acoustico-Verbales (HAV) + », et un groupe témoin, appelé « HAV - ». La réalisation d'une étude en cas-témoin tentait de comprendre pourquoi, chez des patients présentant un même diagnostic clinique de	Redéfinition de la population cible étendu à tout type de trauma.<br><br>Précision apportée sur l'appariement<br><br>Précision donnée concernant la gestion des données permettant de différencier la population exposée au

étude en cas-témoin tentait de comprendre pourquoi, chez des patients présentant un même tableau clinique de TSPT, certains rapportaient la présence d’hallucinations non psychotique et d’autres non.	TSPT, certains rapportaient la présence d’hallucinations non psychotique et d’autres non. Au sein de chacun des deux groupes, une différenciation sera également faite par le type de traumatisme (individuel ou de masse) au travers d’une variable catégorielle binaire.	traumatisme de masse ou traumatisme individuel.
**Page 85 du protocole :**<br><br>**10-L’étude « L’enfant Physalis »**<br><br>**A/ Sélection des sujets**<br><br>• Critères d’inclusion<br><br><u>Groupe HAV +</u><br><br>- Age supérieur ou égale à 8 ans et strictement inférieur à 16 ans lors de l’inclusion<br><br>- Avec HAV (auto-questionnaire de dépistage)<br><br>- Avec un diagnostic de TSPT (MINI-kid 1.1)<br><br>- Affilié(e) à un régime de sécurité sociale ;<br><br>- Ayant une bonne maîtrise de la langue française (francophone)<br><br>Enfants dont les parents ont accepté la participation à l’étude (recueil des consentements éclairés)<br><br><u>Groupe HAV -</u><br><br>- Age supérieur ou égale à 8 ans et strictement	<u>Groupe HAV +</u><br><br>- Age supérieur ou égale à 8 ans et strictement inférieur à 16 ans lors de l’inclusion<br><br>- Avec HAV (questionnaire de dépistage)<br><br>- Avec un diagnostic de TSPT (section « TPST » de la K-SADS-PL)<br><br>- Affilié(e) à un régime de sécurité sociale ;<br><br>- Ayant une bonne maîtrise de la langue française (francophone)<br><br>- Enfants dont les parents ont accepté la participation à l’étude (recueil des consentements éclairés)<br><br><u>Groupe HAV -</u><br><br>- Age supérieur ou égale à 8 ans et strictement inférieur à 16 ans lors de l’inclusion<br><br>- Sans HAV (questionnaire de dépistage)<br><br>- Avec un diagnostic de TSPT (section « TPST » de la K-SADS-PL)<br><br>- Affilié(e) à un régime de sécurité sociale ;	Adaptation du texte en lien avec la modification des outils utilisés pour le dépistage des HAV. Le diagnostic de TSPT est posé à l’aide de la K-SADS-PL et de la MINI-kid 1.1 pour une meilleure intégration de l’étude « L’enfant physalis » intervenant en phase II du protocole « 14.7 » pour les groupe 2, 4 et 5.

inférieur à 16 ans lors de l'inclusion<br><br>- Sans HAV (auto-questionnaire de dépistage)<br><br>- Avec un diagnostic de TSPT (MINI-kid 1.1)<br><br>- Affilié(e) à un régime de sécurité sociale ;<br><br>- Ayant une bonne maîtrise de la langue française (francophone)<br><br>Enfants dont les parents ont accepté la participation à l'étude (recueil des consentements éclairés)	- Ayant une bonne maîtrise de la langue française (francophone)<br><br>- Enfants dont les parents ont accepté la participation à l'étude (recueil des consentements éclairés)	
**Page 86 du protocole :**<br><br>**10-L'étude « L'enfant Physalis »**<br><br>**A/ Sélection des sujets**<br><br>• Critères de non inclusion<br><br>**-** Déficience intellectuelle (QI inf. à 70)<br><br>- Pathologies génétiques, neurologiques ou neurosensorielles<br><br>- Enfant ou adolescent présentant un trouble psychotique ou autistique (MINI-kid 1.1 et section « psychose de la K-SADS-PL)	- Déficience intellectuelle (QI inf. à 70)<br>- Pathologies génétiques, neurologiques ou neurosensorielles<br>- Enfant ou adolescent présentant un trouble psychotique (section « psychose » de la K-SADS-PL) ou autistique (section « trouble du spectre de l'autisme » de la MINI-Kid 1.1)	Adaptation du texte en lien avec la modification des outils utilisés le diagnostic de trouble psychotique posé à l'aide de la K-SADS-PL et de trouble autistique posé à l'aide de la MINI-kid 1.1 pour une meilleure intégration de l'étude « L'enfant physalis » intervenant en phase II du protocole « 14.7 » pour les groupe 2, 4 et 5.

**Page 87 du protocole**<br><br>**10-L'étude « L'enfant Physalis »**<br><br>**C/ Déroulement de l'étude**<br><br>• Intervention<br><br>Tous les patients avec TSPT inclus sont issus du « Programme 14-7 ». L'auto-questionnaire de dépistage des HAV sera proposé aux sujets inclus dans le groupe 2, âgés de 8 à 16 ans, en complément des questionnaires réalisés dans le cadre du « Programme 14-7 ».<br><br>Les sujets dont les HAV non psychotiques ont été dépistés par l'auto-questionnaire constitue le groupe « HAV + » (cas) et le groupe « HAV - » (témoin) comprend les sujets sans HAV non psychotiques et appariés au groupe « HAV + » selon leur sexe et âge.<br><br>Les sujets cas et témoins sont recrutés après information de l'enfant ou de l'adolescent et de sa famille et signature des consentements du patient et des parents (ou représentant de l'autorité parentale).<br><br>La visite d'inclusion (T0) permet, dans un premier temps d'évaluation, de vérifier les critères d'inclusion obtenus par la passation d'échelles réalisées dans le cadre du « Programme 14-7 » :<br><br>- Résultat à l'auto-questionnaire de dépistage des hallucinations acoustico-verbales	L'étude sera proposée à tout patient présentant un TSPT. Une question simple de dépistage des HAV sera proposée aux sujets inclus, âgés de 8 à 16 ans.<br><br>Les sujets dont les HAV non psychotiques ont été dépistés constitue le groupe « HAV + » (cas) et le groupe « HAV - » (témoin) comprend les sujets sans HAV non psychotiques et appariés au groupe « HAV + » selon leur sexe et âge.<br><br>Les sujets cas et témoins sont recrutés après information de l'enfant ou de l'adolescent et de sa famille et signature des consentements du patient et des parents (ou représentant de l'autorité parentale).<br><br>La visite d'inclusion (T0) permet, dans un premier temps d'évaluation, de vérifier les critères d'inclusion obtenus par la passation d'échelles réalisées dans le cadre du « Programme 14-7 » :<br><br>- Résultat au questionnaire de dépistage des hallucinations acoustico-verbales<br>- Diagnostic de TPST posé par la passation de la section « TSPT » de la K-SADS-PL,<br><br>- Elimination du diagnostic de trouble psychotique et autistique par la passation de la K-SADS-PL (section « psychose ») et de la MINI-Kid 1.1 (section « trouble du spectre de l'autisme »)<br><br>- Absence de retard mental évalue à l'aide du Wechsler Intelligence Scale for Children (WISC-V).<br><br>Au terme de cette évaluation, pour les patients correspondant aux critères d'inclusion et ayant donné leur accord pour participer à l'étude, un deuxième temps d'évaluation va permettre la passation des autres tâches	Adaptation du texte en lien avec l'extension des sujets inclus pour tout trauma (de masse et individuel) ainsi que la modification des outils utilisés pour une meilleure intégration de l'étude « L'enfant physalis » intervenant en phase II du protocole « 14.7 » pour les groupe 2, 4 et 5.

- Diagnostic de TPST posé par la passation de la MINI-Kid 1.1,<br>- Elimination du diagnostic de trouble psychotique et autistique par la passation de la MINI-kid 1.1 et de la K-SADS-PL (section « psychose »),<br>- Absence de retard mental évalue à l'aide du Wechsler Intelligence Scale for Children (WISC-V).<br><br>Au terme de cette évaluation, pour les patients correspondant aux critères d'inclusion et ayant donné leur accord pour participer à l'étude, un deuxième temps d'évaluation va permettre la passation des autres tâches spécifiques de l'étude. Cette évaluation peut se dérouler au cours du même entretien, à la suite de la vérification des critères d'inclusion, ou lors d'un deuxième entretien, selon la volonté du sujet.<br><br>Ce deuxième temps d'évaluation comprend :<br><br>- Le recueil des données socio-démographiques et cliniques<br>- La passation des échelles permettant :<br>o L'évaluation de la cognition sociale (NEPSY II)<br>o La détermination du profil émotionnel (EED IV)<br>o L'étude du vécu émotionnel de l'hallucination acoustico-verbale (BAVQ-R), uniquement pour les patients présentant des hallucinations acoustico-	spécifiques de l'étude. Cette évaluation peut se dérouler au cours du même entretien, à la suite de la vérification des critères d'inclusion, ou lors d'un deuxième entretien, selon la volonté du sujet.<br><br>Ce deuxième temps d'évaluation comprend :<br><br>- Le recueil des données socio-démographiques et cliniques<br>- La passation de la MINI-Kid 1.1 pour l'évaluation des comorbidités associées au TSPT : trouble thymiques, troubles anxieux, troubles du comportement, addiction.<br>- La passation des échelles permettant :<br>o L'évaluation de la cognition sociale (NEPSY II)<br>o La détermination du profil émotionnel (EED IV)<br>o L'étude du vécu émotionnel de l'hallucination acoustico-verbale (BAVQ-R), uniquement pour les patients présentant des HAV)<br>-<br><br>A six mois (T1), les patients « HAV + » et « HAV – » ont été revus au cours d'un entretien programmé. Il s'agit :<br><br>- D'évaluer la disparition ou la persistance des hallucinations acoustico-verbales par une nouvelle passation de l'auto-questionnaire de dépistage.<br>- D'effectuer une nouvelle passation de la MINI-Kid 1.1 ou K-SADS-PL (section « TSPT » et « psychose ») afin de mettre en évidence une évolution du diagnostic psychiatrique initialement retenu.<br><br>A un an (T2), le « Programme 14-7 », prévoit un rappel téléphonique de tous les sujets inclus dans l'étude. Le questionnaire d'auto-dépistage des HAV pourra, à ce moment-là, être réalisé par téléphone afin d'évaluer la	

verbales)<br><br>A six mois (T1), les patients « HAV + » et « HAV – » ont été revus au cours d'un entretien programmé. Il s'agit :<br>- D'évaluer la disparition ou la persistance des hallucinations acoustico-verbales par une nouvelle passation de l'auto-questionnaire de dépistage.<br>- D'effectuer une nouvelle passation de la MINI-Kid 1.1 ou K-SADS-PL (section « psychose ») afin de mettre en évidence une évolution du diagnostic psychiatrique initialement retenu.<br><br>A un an (T2), le « Programme 14-7 », prévoit un rappel téléphonique de tous les sujets inclus dans l'étude. Le questionnaire d'auto-dépistage des HAV pourra, à ce moment-là, être réalisé par téléphone afin d'évaluer la persistance des HAV non psychotiques.<br><br>Deux ans (+/- 6 mois) après l'inclusion (T3), le « Programme 14-7 » prévoit un nouveau screening de la population où il sera possible à nouveau possible :<br>- D'évaluer la disparition ou la persistance des hallucinations acoustico-verbales par une nouvelle passation de l'auto-questionnaire de dépistage.<br>- D'effectuer une nouvelle passation de la MINI-Kid 1.1 ou K-SADS-PL (section « psychose ») afin de mettre en évidence une évolution du diagnostic psychiatrique initialement retenu.<br>- D'effectuer une nouvelle passation de la NEPSY II, de	persistance des HAV non psychotiques.<br><br>Deux ans (+/- 6 mois) après l'inclusion (T3), le « Programme 14-7 » prévoit un nouveau screening de la population où il sera possible à nouveau possible :<br>- D'évaluer la disparition ou la persistance des hallucinations acoustico-verbales par une nouvelle passation de l'auto-questionnaire de dépistage.<br>- D'effectuer une nouvelle passation de la MINI-Kid 1.1 ou K-SADS-PL (section « TSPT » et « psychose ») afin de mettre en évidence une évolution du diagnostic psychiatrique initialement retenu.<br>- D'effectuer une nouvelle passation de la NEPSY II, de l'EED IV et de la BAVQ-R (chez les sujets dont les HAV persistent), afin de d'évaluer l'évolution des capacités de cognition sociale et émotionnelle après un TSPT.<br><br>L'étude se termine pour un patient lorsqu'il a achevé les différentes tâches. A la fin de cette étude, les patients poursuivent leur suivi pédopsychiatrique habituel.<br><br>L'étude se déroule sur une période de 3 ans, avec une période d'inclusion de 18 mois et de réévaluation, pour chaque patient inclus, à 6 mois, 1 an et 2 ans.<br><br>Modification du tableau 1 et de la figure 1	

l'EED IV et de la BAVQ-R (chez les sujets dont les HAV persistent), afin de d'évaluer l'évolution des capacités de cognition sociale et émotionnelle après un TSPT.<br><br>L'étude se termine pour un patient lorsqu'il a achevé les différentes tâches. A la fin de cette étude, les patients poursuivent leur suivi pédopsychiatrique habituel.<br><br>L'étude se déroule sur une période de 3 ans, avec une période d'inclusion de 18 mois et de réévaluation, pour chaque patient inclus, à 6 mois, 1 an et 2 ans.		
**Notice d'information et consentement**<br>**L'enfant Physalis V3.0 du 26/07/2019**<br>**Exemple NI destinée au titulaire de l'autorité parentale**<br>**P1 :**<br>Votre enfant a été impliqué lors des événements survenus le 14 juillet 2016, à Nice, de manière directe (présence sur la promenade, par exemple) ou indirecte (présence d'un membre de la famille, par exemple).<br><br>**Objectif de l'étude :** Le but de cette étude est de dépister la présence ou non de symptômes hallucinatoires chez un enfant ayant été impliqué dans les attentats de Nice.	**<u>Version 4.0 du 07/01/2020</u>**<br><br>Ajout de la notion de traumatisme individuel autre que l'attentat du 14 juillet 2016. :<br><br>Votre enfant a été impliqué lors des événements survenus le 14 juillet 2016, à Nice, de manière directe (présence sur la promenade, par exemple) ou indirecte (présence d'un membre de la famille, par exemple) ou a été impliqué à d'autres évènements traumatiques.<br><br>**Objectif de l'étude :** Le but de cette étude est de dépister la présence ou non de symptômes hallucinatoires chez un enfant ayant été impliqué dans les attentats de Nice ou à tout type de traumatisme individuel	Changement de version et date suite à l'amendement<br><br>Ajout des caractéristiques des patients impliqués dans un évènement traumatique à titre individuel<br><br>Les mêmes types de modifications ont été apportés aux notices d'information destinées aux patients
