# Supplementary material for: Relationship between social cognition and emotional markers and acoustic-verbal hallucination in youth with post-traumatic stress disorder: Protocol for a prospective, 2-year, longitudinal case-control study
Source: PLoS One. 2024 Jul 2;19(7):e0306338. doi: 10.1371/journal.pone.0306338 (PMC11218996; doi:10.1371/journal.pone.0306338)
Supplement: Attachment — Submitted filename: Reponse to reviewers.docx [file pone.0306338.s004.docx]

**Dr Louise-Emilie DUMAS - MD**

Service Universitaire de Psychiatrie de l’Enfant et de l’Adolescent

Hôpitaux Pédiatriques de Nice – CHU LENVAL

57 Avenue de la Californie, 06200 Nice

Tel: +334 92 03 03 72/+336 63 92 01 97

Mail: louise-emilie.dumas@hpu.lenval.com

Nice, 16/11/2023

Dear Editor,

Please find enclosed the manuscript revised entitled “Relationship between social cognition and emotional markers and acoustic-verbal hallucination in youth with post-traumatic stress disorder: protocol for a prospective, 2-year, longitudinal case-control study, by Louise‑Emilie Dumas, Arnaud Fernandez, Philippe Auby and Florence Askenazy‑Gittard that we would like to submit in Plos One.

Thank you for reviewing this article and for all your corrections. I have incorporated all your valuable remarks into the text and corrected everything you indicated.

***Reviewer #1:*** *This protocol refers to an interesting and important topic, namely the trajectory of hallucination and psychosis development in adolescents with PTSD. The authors aim to make use of a prospective design to eliminate issues that are currently present in the literature, namely the exclusive use of cross-sectional data. If completed, this study would contribute significantly to elucidating the relationship between PTSD, auditory hallucinations and psychosis development.*

*While relevant and interesting in scope, the study protocol presents with a number of issues that I think are necessary to address:*

1. *Ethics approval/Trial registration: The proposed study is based on a pilot study which was registered under clinicaltrials.gov Since the two studies (proposed (“Physalis”) and Program 14-7) are very different in terms of recruitment (14-7 is already recruited, specifically from people exposed to the attacks) it is not clear to me how these two studies are related. I would suggest the authors make clear in the manuscript; in what way the proposed study is an extension of the original study and why the pilot “Physalis” study had their own clinical trial entry, but this current extension has not.*

*In addition, the clinical trial reference to the program 14-7 study is not helpful, insofar as the recruitment strategy is very different from the proposed study. Furthermore, the amendment and attached study protocol mention the distinction between mass and individual trauma which does not occur in the current manuscript. If they proposed study does not recruit from the Program 14-7 sample at all, this should be made clear and in this case I do not think the current study can be considered an extension of the Program 14-7 study.*

I thank you for your comment which allows me to provide clarification on the “ethical approvals/trial registration” paragraphs. This was to optimize recruitment possibilities for the physalis study. At the beginning of the Physalis study we thought that the 14-7 program would allow for easy recruitment to the Physalis study. Then after a few months, we concluded that it was necessary to open inclusions to all types of trauma and recruitment methods to achieve the number of patients necessary for the study. From February 8, 2020, patients were recruited in the context of the mass trauma of July 14, 2016 but also in the event of individual trauma only.

On a clinical level, the data in the literature do not seem to demonstrate that the type of trauma (mass or individual) can have an influence on the hallucinatory experience.

I replaced part of the paragraph “Informed Consent and Institutional Review Boards” : “*The protocol of the physalis study is ancillary to the protocol "Child and adolescent psychiatry and multidisciplinary research (public health, psychodynamics, neurosciences and human and social sciences) with children exposed to the Nice attack of 14 July 2016 (Program 14-7)". Program 14-7 has the reference number 17-HPNCL-03 and was approved by the Ethics Committee (CE) “Nord-Ouest III” (France) on July 13, 2017 and the National Agency for the Safety of Medicines and Health Products and registered with the National Medicines Safety Agency (ANSM) and all procedures will comply with ethical guidelines international ethics for clinical studies involving human beings. The study protocol has been registered on Clinical Trials.gov (Identifier:NCT03356028).*

*The drafting of the physalis study protocol began in 2018. As the protocol targets patients with PTSD, we moved closer to ongoing studies implicating patients in the attacks of July 14, 2016. An amendment was requested to include the physalis study as an ancillary to program 14-7 and approved by the Ethics Committee (CE) “Nord-Ouest III” (France) on July 27, 2019. In order to facilitate patient inclusion, we have opened the inclusion criteria patients with PTSD to any type of trauma. A new request for amendment was made and approved by the Ethics Committee (EC) “Nord-Ouest III” (France) on February 8, 2020.”*

*2. Study instruments: One of the main variables of interest in this study is the presence of auditory-verbal hallucinations. The authors briefly mention that the instrument was published previously (in a French study, so not accessible to international readers) but give no more detail. The authors should describe this instrument here in detail as well, especially since the outcome variables of this instrument (presence yes/no or a continuous outcome?) is important for the statistical analysis.*

Thank you for your comment, which allows us to specify the tool used in the study. The question, "Have you ever heard a voice in your head that is different from your own and that no one else hears except you?" is drawn from the schizophrenia section of the Diagnostic Interview Schedule for Children (Costello, 1984). The question used in the Physalis study is not part of a standardized tool. The only evaluator of the study deliberately and consistently posed this question to all participants included in the study to ensure the reproducibility of the test. However, it is important to note that this approach may introduce a potential bias into the study, and we will address this in the "Limitations" section of the article.

I added the following sentence in the 'Methods and Analysis' section, subsection 'Assessment and Outcomes Measures,' 'Assessment for Group Allocation': *“**The study evaluator posed the following question to all participants included in the study: “Have you ever heard a voice in your head that is different from your own and that no one else hears except you?” to accurately determine whether they would belong to the “AVH+” or “AVH-“ group."*

I have modified the references associated with the mention of the 'Diagnostic Interview Schedule for Children-Child (DISC-C)' tool.

I also added in “Limitations” the following sentences: *“The question used at patient inclusion to determine their assignment to the “AVH+” or “AVH-“ group was not a standardized tool and could introduce a classification bias. The only evaluator of the study deliberately and consistently posed this question to all participants included in the study to ensure the reproducibility of the test”.*

*3. Sample size calculation: The authors mention that it is impossible to make a quantified hypothesis for the sample analysis, but then go on to provide some information on an analysis anyway. The analysis is missing the effect size that was considered for the calculation. The authors should either state clearly that they have not done a sample size analysis or perform sufficient detail to replicate this analysis (see also the Work of Daniel Lakens regarding sample size justification https://smex-ctp.trendmicro.com:443/wis/clicktime/v1/query?url=https%3a%2f%2fpsyarxiv.com%2f9d3yf%2f&umid=7f3ccb42-b18d-45dc-83dc-5ed85ae75f98&auth=485ed4c97c395527e0d3511589861affbf66eedc-51aecf1c954ca848635a24732b1360850b6ef35d). Additionally, the authors mention the small sample size in their limitations section, however the study is still running. Why not recruit more patients if that’s the main limitation?*

Thank you for this remark which allows me to correct the paragraph "Sample size" with the following sentence: *“Because of the absence of reliable quantified data in the literature, it is impossible to make a quantified hypothesis for the variability of the endpoint. We were consequently unable to calculate a number of subjects necessary for the physalis study”.*

I have also provided additional information in the "Limitations" paragraph with the following sentences modified: *“**The small population sample of the study is due to feasibility. The presence of auditory verbal hallucinations (AVH) in children and adolescents with post-traumatic stress disorder (PTSD) is a rarer symptomatology and more challenging to objectify in clinical interviews compared to the adult population. Previous studies have shown that only 1 to 30% of parents would be informed about hallucinations of their children (60) and only a minority of psychiatrists are aware of these symptoms (60,80,81). Despite our child and adolescent psychiatry department being a reference center for psychotrauma, the number of patients exhibiting this clinical profile remains limited”.*

*4. Data analysis: The data analysis plan is currently incomplete. The authors should mention the specific variables (MINI-KID; NEPSY, etc.) used in each analysis and give more analysis regarding the time point of each analysis. As of now the plan ready as if conducted in a cross-sectional study and it is not clear how the prospective design is considered in the analysis plan. Please include specific analyses regarding the time frame, and mention which analysis is done at which time point (T0, T1, etc.).*

Thank you for your comment, however the data analysis plan is illustrated in Figures 1 and 2 of the article (provided in another document in the submission procedure). These figures precisely describe which analyzes are carried out at each time of the study. But I can also add this in the text of the article with the following paragraph:

- *“At T0, all patients:*
  - *sign a consent to participate in the study after having received informed information,*
  - *pass the questionnaires determining the inclusion criteria in the study: PTSD and psychosis section of K-SADS-PL and the abbreviation form of WISC IV,*
  - *are asked about the presence or absence of AVH to determine their group assignment,*
  - *respond to an interview concerning their socio-demographic data and hallucinatory symptoms,*
  - *respond to the study variables using the NEPSY II, DES IV, BAVQ-R (only for the “AVH +” group) and MINI-Kid questionnaires.*
- *At T1, all patients are reviewed to assess the presence or absence of AVH as well as the evolution of PTSD and psychosis diagnoses (K-SADS-PL) as well as other diagnoses by the MINI-Kid.*
- *At T2, all patients are called by telephone to ask them only about the presence or absence of AVH.*
- *At T3, all patients are reviewed for:*
  - *evaluate the evolution of their diagnosis by passing the PTSD and psychosis section of the K-SADS-PL and Mini-Kid,*
  - *assess the presence or absence of AVH,*
  - *retake the specific study questionnaires: NEPSY II, DES IV and BAVQ-R (if AVH is present).”*

*Additionally, the main analysis seems to be a correlational analysis, however it is not clear to me that all involved variables continuous (especially since the AVH variable is not clearly described in the instruments sections).*

I have included in the appendix of the article a table summarizing all the variables of the study.

Appendix: Description of study variables

| Variable type | Variable name | Variable measurement |
| --- | --- | --- |
| Continuous quantitative variables | Age | years |
|  | Abbreviation form of WISC IV  *Total Intelligence Quotient* | Point |
|  | Differential Emotional Scale IV (DES IV):  *Interest, Joy, Surprise, Anger, Contempt, Disgust, Sadness, Fear, Guilt, Shame, Shyness, and Hostility against self* | From 3 to 15 points/items |
|  | The Beliefs About Voices Questionnaire-Revised (BAVQ-R):   - *Omnipotence,* - *Benevolence,* - *Malevolence,* - *Emotional resistance, Behavioral resistance,* - *Emotional engagement, Behavioral engagement* | From 4 to 24 points/items |
| Categorical variables | Sex | Boy (0) / Girl (1) |
|  | AVH | No (0) / Yes (1) |
|  | PTSD (K-SADS-PL) | No (0) / Yes (1) |
|  | Psychosis (K-SADS-PL) | No (0) / Yes (1) |
|  | MINI-Kid   - *mood disorder: (depression and (hypo)mania);* - *anxiety disorder (panic disorder, agoraphobia, social phobia, obsessive-compulsive disorder and generalized anxiety);* - *behavioral disorder (conduct disorder and attention deficit hyperactivity disorder (ADHD))* | No (0) / Yes (1) |
|  | NEPSY II: « Theory of mind »  *Total Note* | Normal (0)  Lower normal (1)  Lower abnormal (2) |
|  | NEPSY II : «  Emotion recognition »  *Total Note, Happy Error, Sad Error, Neutral Error, Fear Error, Anger Error, Disgust Error* | Normal (0)  Lower normal (1)  Lower abnormal (2) |

*I suspect a between-group analysis (AVH+ vs. AVH-) would be more appropriate.*

This is indeed an inter-group analysis between “AVH +” and “AVH -”. I modified the following sentence in the "Data analysis" paragraph: *“**For the main results, the correlation between the "AVH +" and "AVH -" groups for markers of social cognition (NEPSY II) and emotional markers (EED IV and BAVQ-R) in non-psychotic children and adolescents will be evaluated using correlation analyses”.*

*A correlational analysis also seems not best suited for the relationship between patients’ diagnosis (categorical I assume) and AVH (again, probably a categorical variable).*

Thank you for clarifying this point. I also edited the text to clarify what type of correlation will be achieved between two categorical variables: “*For the correlation of categorical variables, the nullity test of a correlation coefficient will be used (or the Spearman method in the case where the variables do not follow a normal distribution).*”. Apart from the test for the nullity of a correlation coefficient (which needed to be specified in the article), could you tell me which test is the most appropriate in your opinion?

*Here as well, the authors state that correlational analysis is used because of the small sample size, but since this is a study protocol, they can just plan to include more participants if that’s what needed for an appropriate analysis. Overall, this section is most concerning and needs the most comprehensive rewrite.*

As explained previously, the small population sample of the study is due to feasibility. The presence of auditory verbal hallucinations (AVH) in children and adolescents with post-traumatic stress disorder (PTSD) is a rarer symptomatology and more challenging to objectify in clinical interviews compared to the adult population. Despite our child and adolescent psychiatry department being a reference center for psychotrauma, the number of patients exhibiting this clinical profile remains limited.

The study variables have been described earlier. This highlights at least 47 variables at T0 alone. Achieving a multiple regression analysis would require several hundred patients to be included in the study. Recruiting such a large number of patients is not feasible, both due to the rarity of the symptomatology and the chosen methodology, along with the available resources for conducting this study.

*5. Hypothesis: In the discussion section the authors discuss a very interesting theory regarding the co-development of AVH and PTSD symptoms (“dys-sensory perception”), however as far as I can tell they plan no tests or analyses to investigate this model. I suggest the authors deduce specific predictions from this model that can be tested with their collected data.*

Thank you for pointing out this element of the discussion which is also of particular interest to us. The hypothesis of our study in this article is that social cognition and emotional experiences are common markers to explain the presence and persistence of AVH in non-psychotic children and adolescents with PTSD. It is in this hypothesis that we propose the tests and analyzes to study this question. The “dys-sensory perception” model that we suggest is an opening to future transdisciplinary work with multisensory study perspectives and in association with other medical specialties (ENT, etc.)

*Minor concerns:*

*1. Figure quality: The attached figures are not of sufficient quality for publication.*

Thank you for this suggestion which allowed me to rework the quality of figure 1.

*2. Hypotheses: The hypotheses in the introduction should be stated more clearly and in statistical terms.*

Thank you for suggesting that we simply reformulate the hypothesis of the study which I add at the end of the introduction to the article: *“The hypothesis of our study is the social cognitions and emotional markers are cognitive biases associated, or even correlated, with the presence of AVH in children and adolescents with PTSD. This study offers a prospective observation of these markers in the evolution of AVH and psychotic disorder in children and adolescents with PTSD”.*

*3. Data availability: The authors should add a data availability statement to the method section, detailing where their data will be made available.*

The sentence was moved to the article and clarified in method section “Data availability”: *“Study data are collected on a secure computerized database accessible by the study investigator. No datasets were generated or analyzed during the current study. All relevant data from this study will be made available upon study completion”.*

***Reviewer #2:*** *For the sample size calculation, the information that was used to derive the sample size is to be provided e.g. reference of the previous study is to be cited, and whether the sample size calculation in the previous study was performed.*

Thank you for this point also noted by the other reviewer. I modified the paragraph "Sample size" with the following sentence: *“Because of the absence of reliable quantified data in the literature, it is impossible to make a quantified hypothesis for the variability of the endpoint. We were consequently unable to calculate a number of subjects necessary for the physalis study”.*

*The statistical software, its version, publisher name and the accepted level of significance is to be stated.*

Thank you for letting us specify the tool used. The R statistical software was used in this study (R Core Team (2020). R: A language and environment for statistical computing. R Foundation for Statistical Computing, Vienna, Austria. URL https://www.R-project.org /.). It presents a level of significance accepted in scientific literature.

*I add the following sentence and reference to the "Data Analysis" section: “**The R statistical software was used in this study (R Core Team (2020). R: A language and environment for statistical computing. R Foundation for Statistical Computing, Vienna, Austria. URL https://www.R-project.org /.)”*

*The language version for all the tools/inventories/questionnaires including validation information (where applicable) is to be stated.*

Thanks for this suggestion. I have specified all the references of the French versions of each of the tools used in the study in the "Outcomes measured" section of the Methods and analysis section.

*The sentence ‘A bivariate descriptive analysis will look for a significant difference between the case and control groups by the Student's test for the comparison of quantitative variables (or the non-parametric test of Wilcoxon if the validity conditions are not validated), and the* *chi² test (or Fisher's exact test in the case where the validity conditions are not validated) ‘ requires revision.*

Thank you for recommending that I rephrase the following sentence: *“**A bivariate descriptive analysis will look for a significant difference between the AVH+ and AVH- groups. Continuous variables will be compared using the Student's test (or the non-parametric Wilcoxon test if the variables do not follow a normal distribution), and categorical variables with the chi-square test (or the non-parametric bilateral Fisher's exact test if the variables do not follow a normal distribution)”.*

*For Fisher’s exact test, 1 or 2 -tailed test is to be stated.*

In this study, the nonparametric Fisher's exact test is bilateral. The alternative statistical hypothesis H1 is a difference between the AVH+ and AVH- groups and not a value of superiority or inferiority between the groups. In addition, the R software used in the study performs the Fisher's exact test bilaterally by default.

*Chi2 test to be written as* *chi-square test.*

Thank you for your advice, I changed that.

*The statement ‘Pearson's correlation method was used to measure the association between two variables in a small population sample.’ requires revision. Pearson's correlation can be used in small population samples to measure the association between two continuous variables but it is crucial to consider the nature of the data.*

Thank you for your comment, I added the term "continuous" to the following sentence: *"Pearson's correlation method was used to measure the association between two continuous variables in a small population sample”.*

*Typo 'Bonferonni' (Bonferroni)*

Thank you for correcting this error. I changed this in the text.

*The statement ‘Since these are two primary outcomes, correcting the alpha risk by the Bonferonni method is necessary, with alpha = 2.5% for each marker’ could be revised to ‘Since there are two primary outcomes, correcting the alpha risk by the Bonferroni method is necessary, with an adjusted alpha of 2.5% for each marker to control the overall familywise error rate.’*

I thank you very much for this suggestion which I applied in the text.

*Some references did not conform to the journal format.*

I revised the references

*The authors have stated that the limitation of the study is due to the low sample size. This is a good and interesting study and since it is a prospective case-control study, it is a waste not to make an attempt to get more subjects in order to derive more meaningful data/results. Studying the relationship between social cognition and emotional markers and acoustic verbal hallucination in youth with post-traumatic stress disorder requires a good number of subjects. Also, there are many variables that could possibly be involved/interplay, where confounding, mediating, and moderating effects could present in the study and require more meaningful statistical analysis. The authors could make an attempt to increase the sample size by recruiting subjects from other sites. Also, the attrition rates could be more than what the authors have anticipated.*

Thank you for your interest in this work. As we answered previously the small population sample of the study is due to feasibility. The hallucinatory experience in children and adolescents with PTSD is a rarer symptomatology, difficult to differentiate from flashbacks and more challenging to objectify in clinical interviews compared to the adult population. Despite our child and adolescent psychiatry department being a reference center for psychotrauma, the number of patients exhibiting this clinical profile remains limited. We observed a large number of variables in this study for a small number of patients because it is above all an exploratory study which is only the beginning of future, more targeted and multidisciplinary work.

I thank you again for all these corrections that will enable us to significantly improve the overall level of the manuscript.

Sincerely,

Dr Louise-Emilie DUMAS, MD, PhD student
