# Supplementary material for: Relationship between social cognition and emotional markers and acoustic-verbal hallucination in youth with post-traumatic stress disorder: Protocol for a prospective, 2-year, longitudinal case-control study
Source: PLoS One. 2024 Jul 2;19(7):e0306338. doi: 10.1371/journal.pone.0306338 (PMC11218996; doi:10.1371/journal.pone.0306338)
Supplement: Attachment — Submitted filename: Response to reviewers.docx [file pone.0306338.s005.docx]

**Dr Louise-Emilie DUMAS - MD**

Service Universitaire de Psychiatrie de l’Enfant et de l’Adolescent

Hôpitaux Pédiatriques de Nice – CHU LENVAL

57 Avenue de la Californie, 06200 Nice

Tel: +334 92 03 03 72/+336 63 92 01 97

Mail: louise-emilie.dumas@hpu.lenval.com

Nice, 19/02/2024

Dear Editor,

Please find enclosed the manuscript revised entitled “Relationship between social cognition and emotional markers and acoustic-verbal hallucination in youth with post-traumatic stress disorder: protocol for a prospective, 2-year, longitudinal case-control study, by Louise‑Emilie Dumas, Arnaud Fernandez, Philippe Auby and Florence Askenazy‑Gittard that we would like to submit in Plos One.

Thank you for reviewing this article and for all your corrections. I have incorporated all your valuable remarks into the text and corrected everything you indicated.

**Reviewer #1:** I thank the authors for providing a revision and responding politely and extensively to all my comments. The majority of concerns has been addressed. However, there still remain two central issues that I believe should be addressed:

1) Sample size calculation: The authors state that they were unable to calculate a required sample size. Nonetheless they need to include a sample size, so there is a clear criterion for when recruiting should be stopped. Indeed, the authors write that the "number of inclusions necessary was reached on February 8, 2023". If no sample size is defined, this does not make sense. Apparently recruitment has stopped already, but as of now it is unclear why recruiting stopped if no sample size is determined. Since the authors specified their hypothesis I believe the appropriate way of calculating a sample size would be to determine an effect size of interest (how large does the effect in question need to be to be considered relevant?) and then calculating the required number of participants to detect this effect. So, if the authors are able to implement this and adapt their recruiting strategy to the results I would think this is the best action to take. However, I suspect that circumstances might prevent further recruiting (e.g. lack of funding or recruitment possibilities). In that case, the authors should still calculate the required sample size and then state why they are unable to collect this sample transparently (which is something that happens all the time and thus should not be seen as a failure on the authors side but rather an accident of circumstances).

*We thank you for your comment. To clarify, we propose to reinstate this information accompanied by an additional explanation which we hope is satisfactory.*

*We replace the "Simple size" paragraph with the following sentences: “The current literature data does not provide enough information for the calculation of the effect size and the required sample size. We were consequently unable to calculate a number of subjects necessary for the physalis study. We calculated a number of necessary subjects on the feasibility of recruiting and monitoring patients in the child psychiatry department and based on our previous work: a number of 15 patients per group, and a total number of 30 patients in total, were chosen. Considering a rate of loss of follow-up and missing data of around 30%, 20 patients are to be included per arm for a total of 40 patients”.*

2) Data Analysis: The section on data analysis, while updated, is still not sufficiently detailed to allow for replication or meaningful outcomes. I think the issue starts with the hypothesis: "The hypothesis of our study is the social cognitions and emotional markers are cognitive biases associated, or even correlated, with the presence of AVH in children and adolescents with PTSD." First off, I don't understand the distinction between association and correlation drawn here. This should be clarified.

Thank you for your vigilance. We agree with you and corrected accordingly. We removed the distinction between the terms "association" and "correlation".

Second, the "presence of AVH" is a categorical variable in this study, thus the correct analysis to test the hypothesis would a between-subject test (such as t-test) with "social cognitions" and "emotional markers" as outcomes.

We agree with you. Concerning the comparison of the “AVH+” and “AVH-” groups, we made both Student’s test (continuous variables) and chi-square test (categorical variables).

As indicated in the appendix and to clarify this in the article, we changed the "data analysis" paragraph with the following sentences: “*Continuous variables compared using the Student test (or the non-parametric Wilcoxon test if the variables do not follow a normal distribution) concern the emotional markers “DES IV” and “BAVQ-R”. Categorical variables compared using the chi-square test (or the non-parametric two-tailed Fisher exact test if the variables do not follow a normal distribution) concern the social cognition markers NEPSY II "Theory of Mind" and "Emotion Recognition "*”.

The hypothesis should state this clearly, e.g.: "The hypothesis of our study is that social cognitions and emotional markers are more/less strongly expressed in a AVH group than in a similar group without AVH". Ideally, this hypothesis already included the specific names of the variables from the specific measures applied. If the hypothesis is clearly formulated the section on data analysis can be written more clearly as well.

We thank you for your suggestion to rewrite the study hypothesis. We made the following corrections: *“The hypothesis of our study is that social cognitions and emotional markers are expressed differently in the presence or absence of AVH in children and adolescents with PTSD”.*

From my understanding of the collected data the second paragraph in the data analysis section ("A bivariate descriptive analysis will look for a significant difference between the AVH+ and AVH- groups. Continuous variables will be compared using the Student's test (or the non-parametric Wilcoxon test if the variables do not follow a normal distribution), and categorical variables with the chi-square test (or the non-parametric bilateral Fisher's exact test if the variables do not follow a normal distribution.)") seems a sufficient explanation of the testing procedure. The only necessary addition would be a clear written out mention of all variables. For example: "Continuous variables tested with Student's test are: BAVQ-R, DES IV, etc.; Categorical variables assessed with the Wilcoxon test are: NEPSY II Theory of Mind, NEPSY II Emotion recognition, etc.". This should be a direct reflection of the hypothesis stated at the end of the introduction.

Thank you for this suggestion to specify which variables are tested. As explained above, we modify the text with the following sentences: *“Continuous variables compared using the Student test (or the non-parametric Wilcoxon test if the variables do not follow a normal distribution) concern the emotional markers “DES IV” and “BAVQ-R”. Categorical variables compared using the chi-square test (or the non-parametric two-tailed Fisher exact test if the variables do not follow a normal distribution) concern the social cognition markers NEPSY II "Theory of Mind" and "Emotion Recognition ".”*

As the authors state there are only two primary outcomes, I recommend only these two should be listed here. And if exploratory analyses are to be conducted they should be mentioned separately in a section on "exploratory analyses".

This also applies to the section on secondary outcomes. To my understanding this would be a repetition of the first analysis, two years later? This should also be mentioned in the hypothesis so it is obvious what variables are tested specifically.

Thank you for advising us on the reorganization of the “Data analysis” paragraph which allows us to propose the following paragraph:

- *“The main objective*

*In order to answer the study hypothesis, a bivariate descriptive analysis will look for a significant difference between the AVH+ and AVH- groups. Continuous variables compared using the Student test (or the non-parametric Wilcoxon test if the variables do not follow a normal distribution) concern the emotional markers “DES IV” and “BAVQ-R”. Categorical variables compared using the* *chi-square test (or the non-parametric two-tailed Fisher exact test if the variables do not follow a normal distribution) concern the social cognition markers NEPSY II "Theory of Mind" and "Emotion Recognition ". Since there are two primary outcomes, correcting the alpha risk by the Bonferroni method is necessary, with an adjusted alpha of 2.5% for each marker to control the overall familywise error rate.*

- *Exploratory analyses*

*1) A new statistical analysis will be carried out at two years to compare social cognition markers and emotional markers between “AVH+” and “AVH-” groups.*

*2) The correlation between the "AVH +" and "AVH -" groups for social cognition (NEPSY II) and emotional markers (DES IV and BAVQ-R) in non-psychotic children and adolescents will be evaluated using correlation analyses. Pearson's correlation method was used to measure the association between two continuous variables in a small population sample. For the correlation of categorical variables, the nullity test of a correlation coefficient will be used (or the Spearman method in the case where the variables do not follow a normal distribution). Correlations between social cognition and emotional markers and the persistence of AVH in non-psychotic children and adolescents at two years will be reassessed.*

*3) The variables of the groups with or without persistence of AVH at two years will be compared and correlated to evaluate the evolution of the initial diagnosis of PTSD and the development of a psychotic disorder.”*

This also allowed us to correct the writing of the study objectives: *“The main objective of our study protocol is to compare social cognition markers and emotional markers according to the presence or absence of AVH in non-psychotic children and adolescents with PTSD. The secondary objectives of our study protocol are: 1) To compare social cognition markers and emotional markers according to the persistence at 2 years of AVH in non-psychotic children and adolescents with PTSD; 2) To evaluate the correlations between social cognition markers and emotional markers with AVH and with the persistence of AVH over two years; and 3) To evaluate social cognition and emotional markers associated with the evolution of PTSD and the development of psychotic disorder at two years.”*

Minor points:

1) There is no need to repeated the procedure from the figures in detail in the "Procedure" section. Apparently that was a misunderstanding from my first review, for which I apologize.

Thank you for your response to my correction. So we delete the “Procedure” section.

2) The authors replied that they only assess AVH status via a question by the study evaluator. However, in the section "Assessment of group allocation" they still mention a "self-report questionnaire". Please explain what measure is used here.

Thank you for your vigilance. This is a question asked by the assessor and not a self-administered questionnaire. The term “self-report” has been removed from the text.

3) Since the clinical trial registry belongs to the 14-7 program this should be removed.

Thank you for this suggestion. The 14-7 study and the Physalis study are two different research protocols. But as explained in the paragraph " Ethics and dissemination", the Physalis study is an ancillary study to study 14-7 and thus benefits from the same ethical procedure and clinical trial registry.

**Reviewer #2:** The authors have put in great effort to address the comments.

Spearman correlation is suitable for comparison of ordinal data or nonparametric (skewed) data. For comparison of two nominal data, Cramer’s V could be used.

Thank you for your suggestion and we add this statistical analysis in the paragraph “Data analysis”: *"**A Cramer's V test in addition to the chi-square test will be carried out in order to measure the intensity of the relationship of nominal variables between two groups”*.

**Reviewer #3:** I have had the opportunity to review the work "Relationship between social cognition and emotional markers and acoustic-verbal hallucination in youth with post-traumatic stress disorder: protocol for a prospective, 2-year, longitudinal case-control study", the modifications made by the authors have notably improved the previous version and have satisfactorily answered the questions and recommendations of the reviewers. I believe that the article could be ready for publication but, nevertheless, I would like to make some recommendations in case you consider that it can be improved even further.

Regarding the calculation of the effect size, in the event that there is no data from other research on the same characteristics, in addition to pointing this out as a limitation, other research could be used with similar populations (even though different variables have been measured). This is only a suggestion, due to the relevance that reporting the effect size may have.

Thank you for your comment, with which we fully agree. We based ourselves on our previous work studying a similar population to define this necessary number of subjects. We will also be rigorous in our search for studies of similar populations, in order to plan our future work.

As for the statistical analyses, if the authors plan to obtain a small number of participants, the correlational analyzes they may not be the most appropriate and the authors should point out this as a limitation and suggest alternatives adjusted to the expected reality of the data.

Thank you for this suggestion which allows us to adjust the sentence which already mentioned this limit in the article "*The choice of this method for a small sample size remains a limitation in the analysis of the results*".

In this sense, I suggest that they add comparisons between groups of general linear models such as ANOVA, as an alternative.

Thanks for this suggestion. We would have very much liked to have performed an ANOVA analysis in this study if we had compared more than two groups. But in this study, comparisons are only made between two groups, "AVH+" and "AVH-", which excludes the use of ANOVA.

I thank you again for all these corrections that will enable us to significantly improve the overall level of the manuscript.

Sincerely,

Dr Louise-Emilie DUMAS, MD, PhD student
